# How Spatial Heterogeneity of Cover Affects Patterns of Shrub Encroachment into Mesic Grasslands

**DOI:** 10.1371/journal.pone.0028652

**Published:** 2011-12-08

**Authors:** Francesc Montané, Pere Casals, Mark R. T. Dale

**Affiliations:** 1 Forest Sciences Center of Catalonia (CTFC), Sant Llorenç de Morunys, Solsona, Spain; 2 University of Northern British Columbia, Prince George, Canada; University of Northampton, United Kingdom

## Abstract

We used a multi-method approach to analyze the spatial patterns of shrubs and cover types (plant species, litter or bare soil) in grassland-shrubland ecotones. This approach allows us to assess how fine-scale spatial heterogeneity of cover types affects the patterns of *Cytisus balansae* shrub encroachment into mesic mountain grasslands (Catalan Pyrenees, Spain). Spatial patterns and the spatial associations between juvenile shrubs and different cover types were assessed in mesic grasslands dominated by species with different palatabilities (palatable grass *Festuca nigrescens* and unpalatable grass *Festuca eskia*). A new index, called RISES (“Relative Index of Shrub Encroachment Susceptibility”), was proposed to calculate the chances of shrub encroachment into a given grassland, combining the magnitude of the spatial associations and the surface area for each cover type. Overall, juveniles showed positive associations with palatable *F. nigrescens* and negative associations with unpalatable *F. eskia*, although these associations shifted with shrub development stage. In *F. eskia* grasslands, bare soil showed a low scale of pattern and positive associations with juveniles. Although the highest RISES values were found in *F. nigrescens* plots, the number of juvenile *Cytisus* was similar in both types of grasslands. However, *F. nigrescens* grasslands showed the greatest number of juveniles in early development stage (i.e. height<10 cm) whereas *F. eskia* grasslands showed the greatest number of juveniles in late development stages (i.e. height>30 cm). We concluded that in *F. eskia* grasslands, where establishment may be constrained by the dominant cover type, the low scale of pattern on bare soil may result in higher chances of shrub establishment and survival. In contrast, although grasslands dominated by the palatable *F. nigrescens* may be more susceptible to shrub establishment; current grazing rates may reduce juvenile survival.

## Introduction

All ecological systems exhibit heterogeneity and patchiness on a broad range of scales, and this patchiness is fundamental to population dynamics, community organization and element cycling [1]. Since the seminal work of Watt [2], ecologists have been trying to understand the crucial relationship between vegetation pattern and the processes that generate it. Given that plant-plant interactions are local and that plants respond to the so-called “plant's-eye view” of the community [3], [4], the use of space as a surrogate may be an effective way to infer processes from spatial patterns [5].

Woody proliferation into grasslands is a worldwide phenomenon [6] that can cause dramatic changes in community structure and function, such as species diversity and carbon storage [7], [8]. The expansion of woody species in grasslands has been attributed to a number of individual factors, such as climate change, elevated CO_2_ levels, changes in fire frequency, grazing regime, changes in grass competitive ability, and combinations of these factors [9], [10]. These different factors may act as drivers of shrub encroachment across different spatial scales [6], [11]. For instance, whereas factors such as climate or fire [7], [12], [13] may drive shrub encroachment at large scale (regional or landscape scale), interactions between grass species and shrubs [14] may drive shrub encroachment at fine scale. Whether one specific factor driving shrub encroachment acts at large (e.g. climate) or fine scale (e.g. biotic interactions) represents the opposite ends of a continuum of possibilities, with some specific factors (e.g. grazing) acting simultaneously at both large and fine scale. Thus, when selecting forage, herbivores are attracted by grass dominance at the large scale, while they preferentially select grass patches dominated by more palatable species at the fine scale [15].

Overall, all the factors affecting shrub encroachment into grasslands most likely act in a hierarchical manner [16], and if conditions for shrub encroachment are satisfied at large scale (e.g. suitable climate), factors acting at fine scale (e.g. suitable cover types in grasslands) will be essential for precise predictions of shrub encroachment. Thus, it is crucial to understand how fine-scale spatial heterogeneity of cover types in grasslands affects patterns of shrub encroachment. Fine-scale spatial heterogeneity is one of the most conspicuous features of grasslands [17], [18], and this spatial heterogeneity is a major driver of woody plant encroachment [19], [20], [21]. Positive interactions are common in plant communities [22], [23], where protection from herbivores is one of the primary mechanisms by which plants facilitate their neighbours [24]. Facilitation by unpalatable plants, also termed ‘associational resistance’, is therefore considered one of the processes driving woody plant establishment in grasslands [25].

Grasslands in the Pyrenees offer a dramatic example of shrub encroachment [26], [27]. Pyrenean grasslands are usually dominated by patches of grasses with different palatability, which provides an exceptional opportunity to understand how fine-scale spatial heterogeneity of cover types drives the patterns of juvenile shrub encroachment into grasslands. If the ‘associational resistance’ mechanism prevails in these grasslands, we can expect that juvenile shrubs would more frequently appear close to unpalatable grass species (positive spatial associations), and that grasslands dominated by unpalatable species would show more abundant high-density clusters (spatial hot spots) of juveniles. However, in the Pyrenees, shrub encroachment rate seems to be slower in grasslands dominated by unpalatable grass species than in other grassland communities, suggesting that other negative interactions between unpalatable grasses and shrubs may outweigh the potential effects of ‘associational resistance’ [14], [26]. Once juvenile shrubs have established in a site, the effects of grazing and cover types may change with shrub development stage. A detailed spatial analysis of juvenile shrubs at different development stages (e.g. juvenile shrubs of different sizes) and spatial heterogeneity in grasslands dominated by different grass species in grassland-shrubland ecotones may provide valuable insight into the process of shrub encroachment into grasslands.

To address how spatial heterogeneity of cover affects patterns of shrub encroachment, we focused on two mesic grasslands presenting different palatability (i.e. palatable grass-dominated *vs*. unpalatable grass-dominated grasslands) in the Pyrenees. If the ‘associational resistance’ mechanism [25], [28] is important for shrub expansion, we would expect to find a higher number of juvenile shrubs and spatial hot spots in unpalatable-dominated grasslands than in palatable grass-dominated grasslands. In addition, due to grazing pressure, we would also expect that juveniles and unpalatable grasses would frequently appear together (positive spatial associations) while juveniles and palatable grasses would frequently appear less close together (negative spatial associations). As direct assessment of the variables potentially driving shrub encroachment requires long-term experimental manipulations or observations, we use space as a surrogate in an attempt to capture how fine-scale spatial heterogeneity of grasslands affects shrub establishment. Our aim is to understand how spatial heterogeneity of cover affects patterns of shrub encroachment in different grassland communities. This study shows that the use of space as surrogate can be an effective way to understand the processes taking place in spatially heterogeneous environments, such as shrub encroachment into spatially heterogeneous grasslands.

## Materials and Methods

### Ethics statement

All necessary permits were obtained for the described field studies. The authority responsible for the Alt Pirineu Natural Park issued the permission for our field studies.

### Study site

This research was conducted in Collada de Montalto, Campirme (42°37′47″N, 1°11′15″E; 2100 m a.s.l.), a mountain mesic site in the Pyrenees located in the Alt Pirineu Natural Park (Catalonia, Spain). Mean annual and winter temperature are 2.5°C and −3°C, respectively, and mean annual precipitation is 1397 mm based on the closest meteorological station to the study site (Boí, 42°27′58″N, 0°52′22″E; 2540 m. a.s.l.). The area is usually under snow from December to April. Soils at the site develop over slates, and the soil profile is approximately 60–80 cm deep.

The study site is a gentle south-facing slope. Vegetation is a mosaic of grassland and shrubland. The mesic grasslands are dominated by patches of *Festuca nigrescens* Lam. or *Festuca eskia* Ramond ex DC., although other species are present, including *Calluna vulgaris* (L.) Hull, *Carex sp.* and *Juniperus communis* L. *F. nigrescens* grows mostly in mesic grasslands while *F. eskia* grows in a wide range of subalpine and alpine grassland types, from steep slopes to mesic grasslands. In our site, both these grasses coexist without apparent differences in soils under *F. nigrescens* or *F. eskia* patches.

Studies documenting shrub expansion in Pyrenean grasslands [27] have identified the legume *Cytisus balansae* ssp *europaeus* (G. López & Jarvis) Muñoz Garmendia as one of the most abundant shrubs encroaching on this area's montane and subalpine grasslands. Historical aerial photographs confirm that shrubland surface has increased at the site and that shrub encroachment has been taking place since the mid-20th century. Both clonal growth from roots and seed dispersal play important roles in the spread of *Cytisus* into grasslands [29]. Despite *Cytisus* being a widespread shrub in southern-European mountain grasslands, little is known about the mechanisms and consequences underpinning *Cytisus* encroachment in different grassland communities. Over the last few decades, the site has been grazed by cattle, horse and sheep, which usually graze in *F. nigrescens* patches, at a grazing rate of approximately 80–90 livestock unit grazing days (LUGD) ha^−1^ (Taull M., pers. comm. 2009). Domestic herbivores graze strongly on *F. nigrescens* but ignore *F. eskia* and adult *Cytisus* due to their unpalatable characteristics [29], [30]. *Cytisus* is a leafless legume shrub and a shade-intolerant species that spreads in sites with low overstory cover [31]. Its lifespan is approximately 30–40 years. Although shepherds in the Pyrenees have traditionally used burning as a management tool for transforming encroached land to grassland, the site has not been burned for at least 30 years (Forest Office Pallars Sobirà, pers. comm. 2007).

### Plot selection and field sampling

In summer 2008, we randomly picked eight plots (20×10 m) in grassland-shrubland ecotones from 368 ha. Half of the plots were located in *F. eskia*-dominated grasslands while the other half were located in *F. nigrescens*-dominated grasslands. Although both *F. eskia* and *F. nigrescens* coexisted in most of the plots, only the species that showed the greatest percentage cover in the plot was considered dominant (approximately 50%; Table 1). For each grassland type, two plots were placed in areas where shrub encroachment was occurring in an uphill direction, and two plots were placed in areas where shrub encroachment was occurring in a downhill direction. We elected to sample in different encroachment directions due to the fact that shrub seed dispersal by gravity is limited upslope but favoured downslope, thus modifying encroachment rates. However, since juveniles encroaching upslope and downslope both showed similar counts and spatial patterns, the plots with different encroachment directions in the same grassland community were combined for statistical analysis. Thus, we contrasted two experimental conditions (*F. nigrescens* and *F. eskia* grasslands) with four plots per experimental condition. In each rectangular plot, the 10-m sides were located following maximum slope direction (slope = 10–25%), whereas the 20-m sides of the plots were located following contour lines (slope = 0). Thus, one of the 20-m sides bordered onto shrubland while the other side bordered onto open grassland. We chose 20×10 m plots because in a preliminary survey, neither adult nor juvenile shrubs were found at more than 10 m distance from shrubland boundary, and also to ensure a minimum number of 30 juveniles per plot. Adult shrubs covered less than 25% of the plot surface area.

**Table 1:** Total number of juvenile *Cytisus* shrubs in each plot and total cover (%) for each cover type (species, bare soil and litter).

	*Festuca nigrescens* grassland				*Festuca eskia* grassland			
	Uphill		Downhill		Uphill		Downhill	
	NigUp1	NigUp2	NigDown1	NigDown2	EskUp1	EskUp2	EskDown1	EskDown2
Number of juvenile *Cytisus* shrubs	130	64	76	120	42	122	48	70
*Cover (%)*								
Adult *Cytisus* shrubs	15.5	18.7	21.8	18.8	16.3	18.9	16.9	21.2
*Festuca nigrescens*	61.1	55.5	53.8	50.9	10.1	3.2	4.5	15.4
*Festuca eskia*	-	12.3	-	15.4	54.5	55.8	50.9	47.1
*Calluna vulgaris*	1.2	-	-	4.3	0.5	3.2	1.0	0.1
*Carex sp.*	10.8	11.8	3.3	3.6	0.1	0.8	2.0	2.8
*Juniperus communis*	0.3	-	0.9	-	-	-	-	-
Litter	6.4	1.1	6.7	3.8	14.0	16.6	20.6	10.6
Bare soil	4.7	0.6	13.5	3.2	4.5	1.5	4.1	2.8

Plot codes: NigUp: plot dominated by *F. nigrescens* with shrubs encroaching uphill; NigDown: plot dominated by *F. nigrescens* with shrubs encroaching downhill; EskUp: plot dominated by *F. eskia* with shrubs encroaching uphill; EskDown: plot dominated by *F. eskia* with shrubs encroaching downhill.

To assess fine-scale effects on shrub establishment, each plot (20×10 m) was gridded into 3200 25×25-cm quadrats. A 1-m vegetation sampling frame divided into sixteen 25×25-cm squares was used to sample all the quadrats in each plot. In each quadrat, we measured the cover of *F. nigrescens*, *F. eskia*, *Calluna vulgaris*, *Carex sp.*, *Juniperus communis*, litter and bare soil in different cover classes (0%, 1%–25%, 26–50%, 51%–75% or 76%–100%). Cover types were only considered if they accounted for at least 10% cover within a quadrat. In addition to grid sampling, we also measured the coordinates of all juvenile shrubs. As the study focus is grassland encroachment, only juvenile shrubs that had a minimum distance of 20 cm to the closest adult shrub were measured and mapped. Height and diameter of each juvenile shrub were measured using a ruler and a digital caliper, respectively. According to diameter-age relationships for *Cytisus*, the ages of juveniles in the plots ranged between 0 and 6 years.

### Spatial patterns of juveniles

We considered the total number of juveniles in each plot. In addition, two size classes of juvenile *Cytisus*, i.e. short (height<10 cm) and tall (height>30 cm), were used as indicators of different shrub development stages. For each plot, the total number of juveniles and the number of juveniles in different development stages (short and tall) were compared between different grassland communities using ANOVA.

Spatial hot spots of juvenile shrubs (high-density clusters of juvenile shrubs in a particular plot) were detected using local spatial statistics [32], [33]. In order to detect spatial hot spots of juveniles, the local *G_i_* ^*^ statistic [34] was calculated for juvenile shrub counts in each 25×25-cm quadrat in a given plot, using the equation:
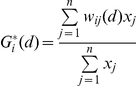
where *w_ij_(d)* expresses the binary connections based on distance (1.5 units  = 37.5 cm) between quadrats *i* and *j* in a plot, and *x_j_* denotes the counts of juvenile shrubs in quadrat *j*. A significant *G_i_* ^*^ value (*p*<0.05) reveals hot spots of juveniles. An ANOVA was run to test for differences in the number of hot spots between different grassland communities. The spatial hot spots were calculated using PASSaGE v2 software [35].

### Scale of pattern of cover types and spatial associations between juvenile shrubs and covers

The scales of heterogeneity in a landscape detected by pattern analysis are important characteristics of the landscape, as they affect the responses of organisms to their environment and other ecological processes [32]. The scale of pattern is defined as half the average distance between patch centres [36]. The scale of pattern of each cover type (plant species, litter and bare soil) in the plots was analyzed using four-term local quadrat variance, 4TLQV [36], an extension of the two-term local quadrat variance (TTLQV) method used to analyze scale of pattern in one-dimensional transects. The local variance in 4TLQV is calculated based on the total cover in each of four mutually contiguous square blocks, each consisting of 1, 4, 9, 16… of the original sample quadrats. The original quadrats are combined into square blocks, which also form a square of four blocks for 4TLQV. The variance is essentially the squared difference between the total cover in one block and the average total cover in the adjacent three blocks. This calculation is then performed for a range of block sizes. Peaks in 4TLQV indicate the scale of pattern in the data. To confirm that these peaks did not occur by chance, randomization tests were performed to construct a null variance model and the associated 95% confidence intervals.

Associations between juvenile shrubs and the different cover types were assessed using four-term local quadrat covariance, 4TLQC [36], a method similar to 4TLQV but which examines covariation between variables of interest in quadrats. The local covariance in 4TLQC is calculated based on the abundance of juveniles and the total cover of a particular cover type in each of four mutually contiguous square blocks, each consisting of 1, 4, 9, 16… of the original sample quadrats. The original quadrats are combined into square blocks, which also form a square of four blocks for 4TLQC. The covariance calculation is similar to the variance calculation described above for 4TLQV, but instead of squaring the difference of one variable (e.g. total cover for one cover type), the difference in the pair of variables (e.g. abundance of juveniles and total cover for one cover type) is multiplied. This calculation is then performed for a range of block sizes. Peaks in this covariance are indicative of scale of spatial association between juveniles and a particular cover type (being positive when juveniles and a particular cover type tend to be found together and negative in the opposite case). To confirm that these peaks did not occur by chance, randomization tests were performed to construct a null covariance model and the associated 95% confidence intervals. Peaks with either positive or negative values greater in absolute magnitude than the confidence limits were interpreted as indicative of positive or negative associations.

In addition, to test whether the spatial associations between juveniles and different cover types changed at different shrub development stages, we assessed the spatial associations (4TLQC) between the different cover types and all short (height<10 cm) and tall (height>30 cm) juveniles in a particular plot. Both scale of pattern and the spatial associations between the different cover types and juveniles were calculated using PASSaGE v2 software [35].

### Relative Index of Shrub Encroachment Susceptibility (RISES)

The associations between juveniles and each cover type were summarized to evaluate the chances of shrub encroachment for a given plot, using a new index, “Relative Index of Shrub Encroachment Susceptibility” (RISES). Although a given 4TLQC analysis can find significant peaks at different block sizes, RISES is based on the first significant peak (i.e. the one at the smallest block size). RISES was calculated for each plot using the following equation:
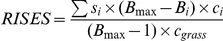
For each cover type (*i*) in a particular plot, 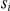 takes the value +1 or −1 depending on whether cover type *i* was positively or negatively associated with *Cytisus*. When no association was found between *Cytisus* and cover type *i*, 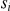 takes the value 0. 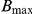 is the maximum block size (expressed as number of quadrats) used in the 4TLQC calculations, whereas 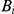 is the smallest block size (expressed as number of quadrats) in which 4TLQC found a significant association. 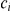 is the cover (%) for the cover type *i* in the plot, while 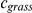 is the cover in % for the grassland surface in the plot, and 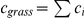. RISES summarizes the results of the covariance analyses by creating a weighted sum of the scales at which the first significant (positive or negative) peaks are found in plots of covariance as a function of block size (scale). The index takes values close to +1 when there are only strong positive associations with all cover types, and values close to −1 when there are only strong negative associations found. Values close to 0 can indicate either a mixture of positive and negative associations, or a neutral susceptibility to shrub encroachment. RISES values are “by definition” significant, because the term 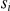 ensures that only significant spatial associations (based on the null model and 95% confidence intervals) are included.

There are many predictive habitat distribution models in ecology [37], but most of them do not incorporate biotic interactions terms (e.g. competition, facilitation) into spatial modelling [38]. The better predictions for biotic interactions can be integrated using indirect variables, such as cover type, for modelling at small spatial scales [37]. Therefore, the new RISES index, which integrates biotic interactions based on covariation of spatial patterns, may help fill this gap. As the RISES index is based on results of 4TLQC spatial analysis, it allows us to integrate the sign of spatial associations and the spatial scales at which these spatial associations occur, which is something that could not be achieved with an index based, for example, on contingency tables using presence/absence data.

Although this index does have its drawbacks (for instance, it assumes that juvenile shrubs have the same chance of establishing in a particular cover type regardless of proximity to adult shrubs), RISES makes it possible to assess potential vulnerability of grassland communities to shrub encroachment based on the surface area of different cover types and the spatial associations (strength and sign) between the different cover types (plant species, litter and bare soil) and juvenile *Cytisus*. Thus, grasslands with the same proportion of cover types may have different RISES values if there are distinct spatial associations between cover types and juveniles. RISES can be computed on any other plot of contiguous quadrats for which the cover or abundance of each cover type is known. RISES can be used not only to assess shrub encroachment into grasslands but also to assess invasibility in spatially heterogeneous habitats by a non-native plant species or in any other setting where there are signicant patterns of spatial covariance between species or cover types.

## Results

### Spatial patterns of juvenile shrubs

**Figure 1:**
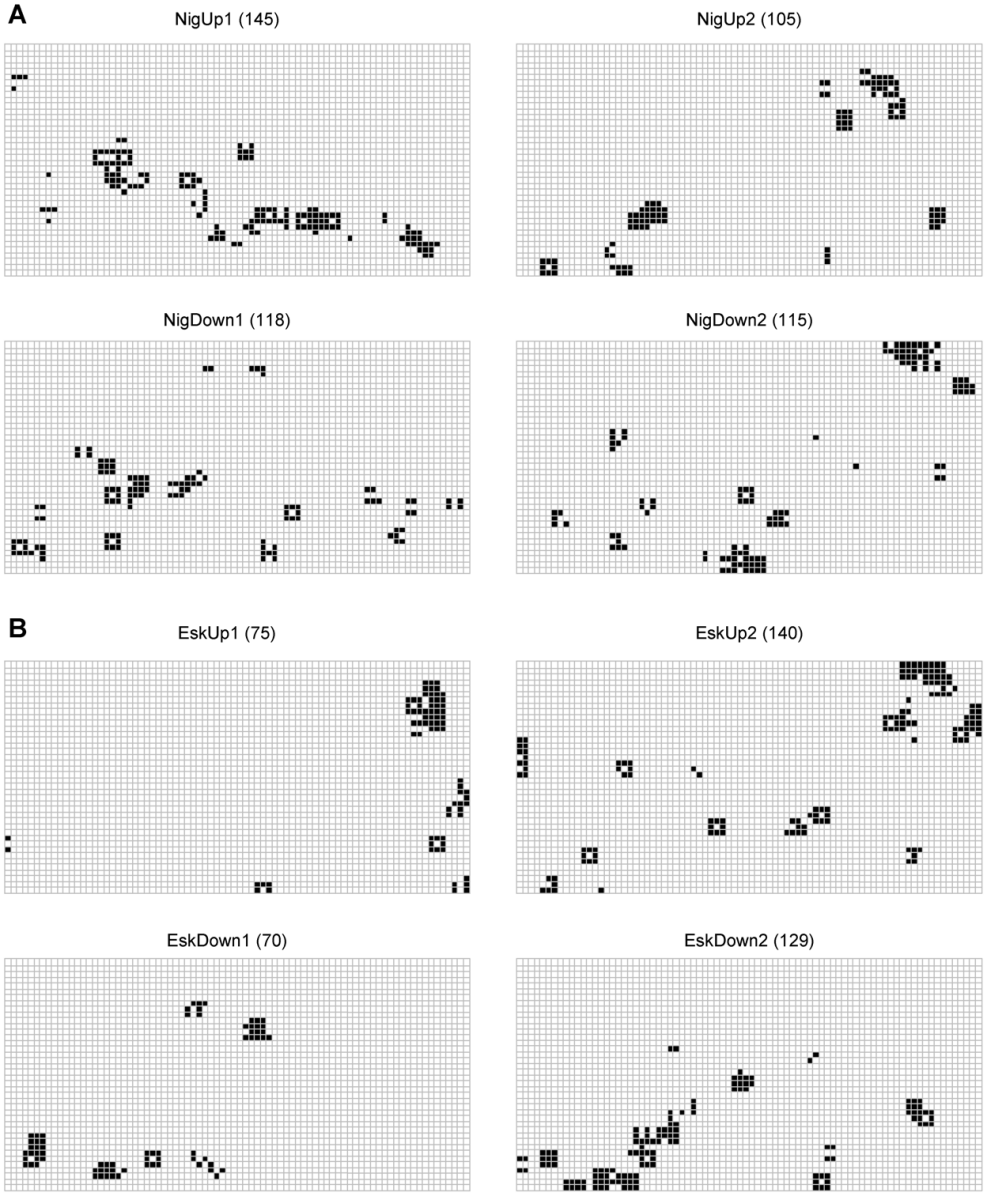
Juvenile *Cytisus* hot spots detected with local *G_i_* ^*^ statistic in *F. nigrescens* or *F. eskia* grasslands. Number of hot spots is given in brackets.

The total number of juvenile shrubs found in the plots was similar between different grassland communities (*p* = 0.400), with values ranging from 42 to 130 juvenile shrubs per plot (Table 1). However, the number of short (height<10 cm) juvenile shrubs per plot was greater in *F. nigrescens* than *F. eskia* grasslands (45.0 *vs.* 17.3 juveniles per plot; *p* = 0.049). In contrast, total number of tall (height>30 cm) juvenile shrubs per plot was lower in *F. nigrescens* than *F. eskia* grasslands (1.2 *vs.* 9.2 juveniles per plot; *p* = 0.023). Number of spatial hot spots was not significantly different between grassland communities (*p* = 0.421; Figure 1). However, *F. nigrescens* plots always had over 100 hot spots while two *F. eskia* plots had a hot spot count of less than 80.

### Scale of pattern of cover types and spatial associations between juvenile shrubs and cover types

**Figure 2:**
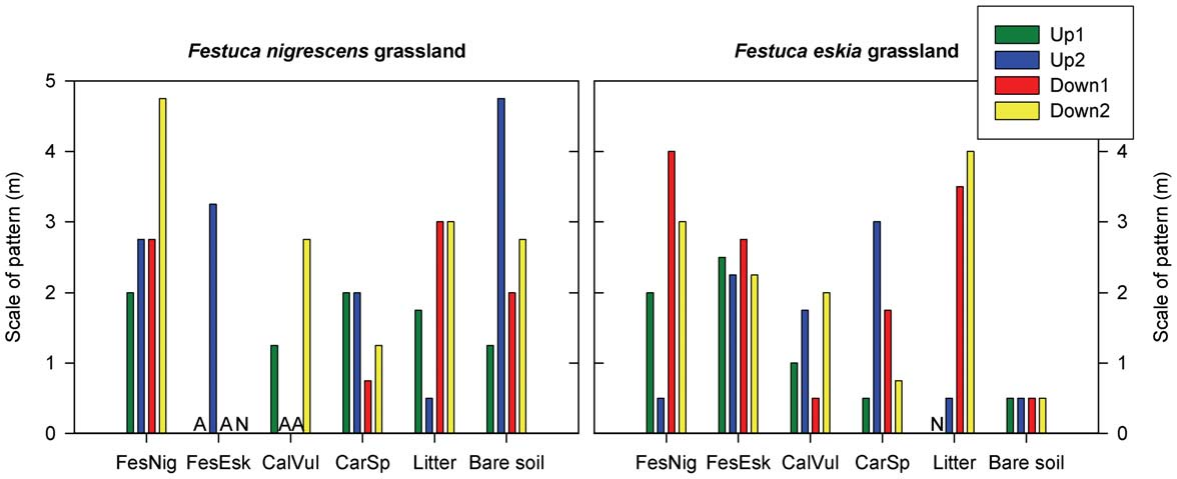
Scale of pattern for each cover type in *F. nigrescens* or *F. eskia* grasslands. Cover types: **FesNig** = *Festuca nigrescens*; **FesEsk** = *Festuca eskia*; **CalVul** = *Calluna vulgaris*; **CarSp** = *Carex sp.* (**A** = cover type absent in the plot; **N** = scale of pattern is non-significant below 5 m).

In each grassland type, the dominant grass species cover was approximately 50%. The highest litter cover values were found in *F. eskia* plots. Bare soil cover was similar between grassland types (Table 1). The most important difference was found in the scale of pattern of bare soil depending on grassland community (Figure 2): whereas bare soil showed a scale of pattern at 0.5 m in most of the *F. eskia* plots, its scale of pattern in the *F. nigrescens* plots was far more variable among plots (from 1.25 to 4.75 m). The remaining cover types did not show clear trends (Figure 2).

**Figure 3:**
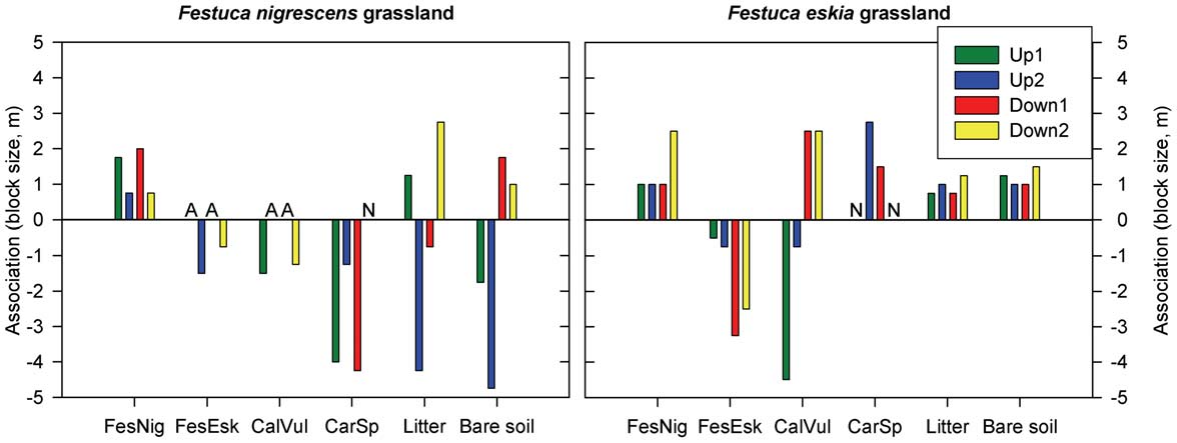
Scales of positive and negative associations between juvenile *Cytisus* and each cover type in *F. nigrescens* or *F. eskia* grasslands. Cover type: **FesNig** = *Festuca nigrescens*; **FesEsk** = *Festuca eskia*; **CalVul** = *Calluna vulgaris*; **CarSp** = *Carex sp.* (**A** = cover type absent in the plot; **N** = association is non-significant below 5 m). All the scales of association except those indicated (**N**) are significant (*p*<0.05) based on randomization tests used to build a null covariance model and the associated 95% confidence intervals.

Regardless of grassland community, *F. nigrescens* and *F. eskia* grass patches showed opposite associations with juvenile *Cytisus* (Figure 3; App. 1). Positive associations were found with *F. nigrescens* (scales between 0.75 and 2.5 m) while negative associations were found with *F. eskia* (from 0.5 to 3.25 m). For the remaining cover types, we found contrasting associations with juvenile *Cytisus* depending on grassland type (Figure 3). In *F. nigrescens* grasslands, most of the non-dominant cover types showed negative associations, except litter and bare soil which did not show clear association trends (Figure 3). In contrast, in *F. eskia* grasslands, most of the non-dominant cover types showed positive associations, except *Calluna vulgaris* which showed both positive and negative associations, and *Carex sp.* which showed a positive association in just two out of four plots. In *F. eskia* grasslands, litter and bare soil always showed positive fine-scale associations (from 0.75 to 1.25 m for litter and from 1.0 to 1.5 m for bare soil; Figure 3).

**Figure 4:**
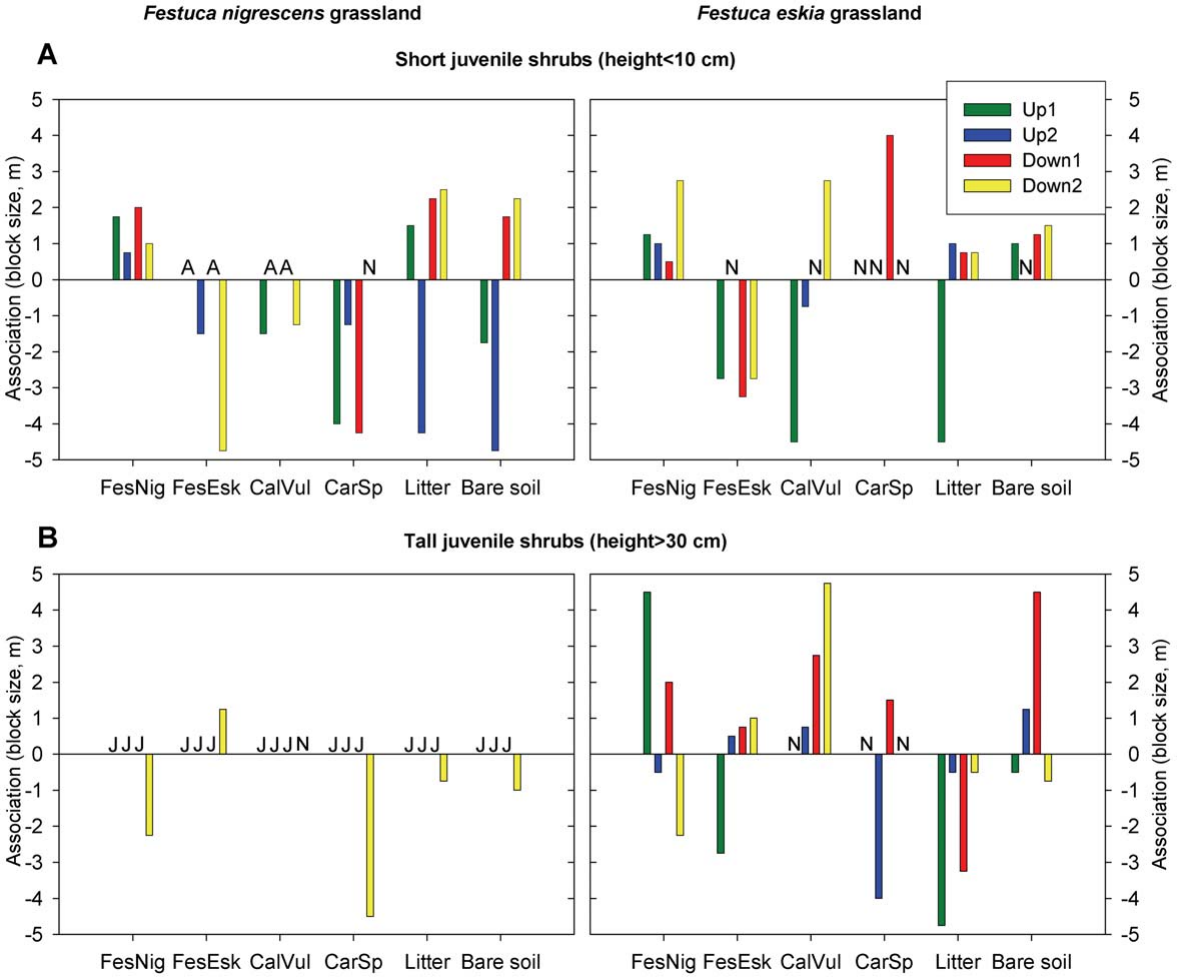
Scales of positive and negative associations between each cover type and a) short juvenile *Cytisus* (height<10 cm) and b) tall juvenile *Cytisus* (height>30 cm) in *F. nigrescens* or *F. eskia* grasslands. Cover types: **FesNig** = *Festuca nigrescens*; **FesEsk** = *Festuca eskia*; **CalVul** = *Calluna vulgaris*; **CarSp** = *Carex sp.* (**A** = cover type absent in the plot; **J** = juvenile *Cytisus* with height>30 cm absent in the plot; **N** = association is non-significant below 5 m). All the scales of association except those indicated (**N**) are significant (*p*<0.05) based on randomization tests used to build a null covariance model and the associated 95% confidence intervals.

Short juvenile shrubs showed opposite associations with *F. nigrescens* and *F. eskia* grasses, regardless of grassland community (Figure 4). Positive associations were found with *F. nigrescens* (scales between 0.5 and 2.75 m) while negative associations were found with *F. eskia* (from 1.5 to 4.75 m). In contrast with the associations found for short juvenile shrubs, tall juvenile *Cytisus* showed fairly positive associations with *F. eskia* (except in one plot) and unclear associations with *F. nigrescens* (negative associations in three out of five plots; Figure 4). In addition, only very few plots in *F. nigrescens*-dominated grasslands contained tall (height>30 cm) juveniles (Figure 4).

### Relative Index of Shrub Encroachment Susceptibility (RISES)

The RISES index indicated opposite susceptibility to shrub encroachment between *F. nigrescens* and *F. eskia* grasslands (*p* = 0.002). Mean RISES values were higher in *F. nigrescens* grasslands than *F. eskia* grasslands (+0.43 *vs.* −0.18, respectively).

## Discussion

### Susceptibility of grassland communities to shrub establishment

Total number of juvenile shrubs at early development stage (i.e. height<10 cm) was greater in grasslands dominated by the palatable *F. nigrescens* than in grasslands dominated by the non-palatable *F. eskia*. *F. eskia* grass patches and juveniles were negatively associated (see Appendix S1), suggesting that ‘associational resistance’ plays a less relevant role than other negative mechanisms driving *Cytisus* shrub establishment. In contrast, *F. nigrescens* was positively associated with juvenile *Cytisus* (see Appendix S1). These associations cannot be due to differences in edaphic factors, since both *F. eskia* and *F. nigrescens* species appear in similar soils that greatly differ from the soils occupied by other grassland communities in the Pyrenees, such as *Festuca paniculata* grasslands [14]. Therefore, the associations found between both *Festuca* species and juvenile *Cytisus* still require experimental evidence and identification of the mechanisms driving them. *F. eskia* may exert stronger belowground competition with shrubs than other grass species [39], since being ungrazed it can allocate resources mainly to belowground biomass and develop a deeper rooting system than other grass species [40]. In contrast, the fact that *F. nigrescens* invests in resprouting after grazing may result in slower belowground biomass growth [41], which may favour shrub establishment. Furthermore, aboveground constraints (e.g. light) may also explain the associations found [42]. Since grazing pressure is greater in *F. nigrescens* patches than in *F. eskia* patches, our results run opposite to what would be predicted by classic indirect plant-herbivore studies [24], [25].

Looking at the RISES values, *F. nigrescens*-dominated grasslands appear more susceptible to shrub encroachment than *F. eskia*-dominated grasslands. Thus, shrub establishment into *F. eskia*-dominated grasslands may primarily be constrained by the abundance of secondary cover types having positive associations with juveniles, such as *F. nigrescens* patches, litter or bare soil. The role of these secondary cover types must not be overlooked in either grassland community (see Appendix S2 for examples of changes in RISES values with secondary cover types). Interestingly, although *F. nigrescens* and *F. eskia* patches associations with *Cytisus* were similar in both grassland types, there was a general drift for the rest of the cover types, from mainly negative associations in *F. nigrescens*-dominated grasslands to mostly positive associations in *F. eskia*-dominated grasslands. For instance, associations between *Calluna vulgaris* or *Carex sp.* and juvenile *Cytisus* were mainly negative in *F. nigrescens* grasslands yet mostly positive or neutral in *F. eskia* grasslands, suggesting the existence of indirect interactions in these communities [24]. These association drifts might be related to alterations of competitive effects between species, and may thus determine species assemblages. Litter and bare soil also showed positive and strong associations with juveniles in *F. eskia* grasslands (see Appendix S1) but unclear effects in *F. nigrescens* grasslands. Grass litter plays a complex role in plant establishment [43], and our results may also be partially explained by the different physical and chemical properties of litter [44]. Bare soil has significant and diverse effects on the establishment of woody plants [45], [46]. Either due to intrinsic community structure or to factors such as grazing and/or trampling, grassland communities show different bare soil patterns which may, in turn, affect shrub establishment. Based on our results, the lower scale of pattern on bare soil in *F. eskia* than in *F. nigrescens* grasslands may offer major chances of shrub establishment and survival, with juveniles on bare soil being protected by surrounding tall and unpalatable *F. eskia* tussocks.

### Differential shrub encroachment into grasslands

Although RISES values together with the number of juveniles at early development stage (height<10 cm) showed that *F. nigrescens* grassland was a more susceptible habitat for shrub establishment than *F. eskia* grasslands, the number of juvenile shrubs at late stage of development (height>30 cm) was lower in *F. nigrescens* than *F. eskia* grasslands. This may be explained by the shifts from positive to negative associations between these two dominant grass species (*F. nigrescens* and *F. eskia*) and juvenile shrubs depending on development stage [47]. Assuming that the timeframe for juvenile establishment into grasslands (6 years) is short enough to avoid major changes of cover types in time, our results suggest that unpalatable *F. eskia* patches seem to limit *Cytisus* establishment (e.g. plant emergence) but improve the survival of established shrub juveniles. In contrast, palatable *F. nigrescens* patches seem to favour *Cytisus* establishment but diminish the survival of established juvenile shrubs. Given that one of the most apparent differences between these two grasslands is the palatability of the dominant grass, we suggest that the current grazing regime slows down *Cytisus* encroachment into grasslands dominated by the palatable grass *F. nigrescens*. Differences in juvenile survival between grassland communities may also partly explain the similar number of spatial hot spots found in both grassland types, with the highest survival of juveniles in *F. eskia* grasslands counterbalancing the lower susceptibility to shrub encroachment in these grasslands. There are several reports of mortality rates of various plant species decreasing significantly as abundance of neighbouring unpalatable species increases [48], [49]. Thus, associational resistance in *F. eskia* patches may not appear in the first development stages of *Cytisus* but instead emerge later on, thus increasing the survival of juvenile *Cytisus*. There is previous evidence of ontogenetic effects on grass-woody interactions [50], and these ontogenetic constraints are often critical in understanding and managing plant populations where particular life stages require specific conditions not shared by other life stages [47].

### Conclusion

Our study suggests that fine-scale spatial heterogeneity of cover types is a crucial factor in shrub encroachment into grasslands. *Cytisus* shrub encroachment into mesic grasslands is constrained first by the chances of new individuals being able to establish, and second by the success of their expansion. Shrub establishment is shaped by spatial patterns of suitable cover types. Grasslands dominated by *F. nigrescens* are more susceptible to shrub establishment than grasslands dominated by *F. eskia*. In *F. eskia* grasslands, the strong interspecific competition between *F. eskia* and *Cytisus* may reduce the number of juvenile shrubs at early development stage compared with *F. nigrescens* grasslands. However, the low scale of pattern of bare soil in *F. eskia* grasslands may offer a major opening for shrub establishment and survival in these grasslands. The associational resistance mechanism may act not at the establishment phase but later on, increasing the survival and proliferation of juvenile shrubs into unpalatable *F. eskia* grasslands, while current grazing rates may reduce the success of proliferation in the more palatable *F. nigrescens* grasslands, despite them being more susceptible to shrub establishment.

## Supporting Information

Appendix S1 **Plots of the main associations between juvenile shrubs and different cover types in grasslands.** Plots of the main associations between juvenile *Cytisus* (empty circles) and different cover types in *F. nigrescens*- and *F. eskia*-dominated grasslands. Cover in the plots was measured in 25×25 cm quadrats. The sign of the association (positive or negative) is given in brackets.
(PDF)

Appendix S2 **Changes in RISES values in different grasslands.** Changes in RISES values in a) *F. nigrescens-*dominated grasslands and b) *F. eskia*-dominated grasslands with dominant species cover ranging from 50 to 90% and an additional represented non-dominant cover type ranging from 0 to 40%. Grassland cover (C_grass_) was assumed to be 100% in all the examples. For each case, an equally-distributed total surface cover of 10% was assumed for the rest of the non-represented cover types (e.g. *Carex sp*, litter, etc.) in all the situations, and the spatial association values used were derived from our plots. According to the results obtained, changes in surface cover of the dominant grass species are not enough to predict changes in RISES, as RISES values are also dependent on surface cover of non-dominant cover types in both *F. nigrescens-* and *F. eskia-*dominated grasslands.
(PDF)
