# Supplementary material for: How Spatial Heterogeneity of Cover Affects Patterns of Shrub Encroachment into Mesic Grasslands
Source: PLoS One. 2011 Dec 8;6(12):e28652. doi: 10.1371/journal.pone.0028652 (PMC3234287; doi:10.1371/journal.pone.0028652)
Supplement: Appendix S1 — Plots of the main associations between juvenile shrubs and different cover types in grasslands. Plots of the main associations between juvenile Cytisus (empty circles) and different cover types in F. nigrescens- and F. eskia-dominated grasslands. Cover in the plots was measured in 25×25 cm quadrats. The sign of the association (positive or negative) is given in brackets. (PDF) [file pone.0028652.s001.pdf]

Appendix S1

NigUp1

a) *F. nigrescens* (+)

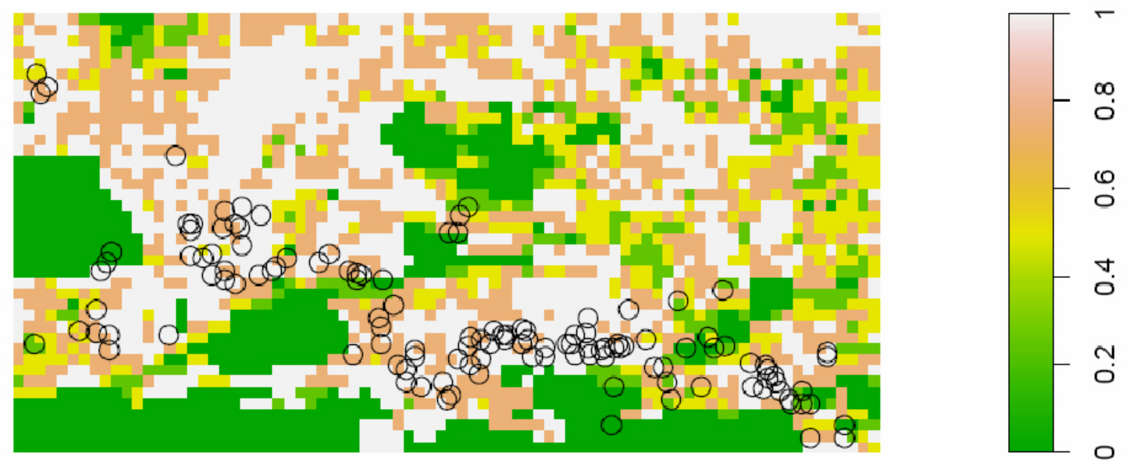

b) *Calluna vulgaris* (-)

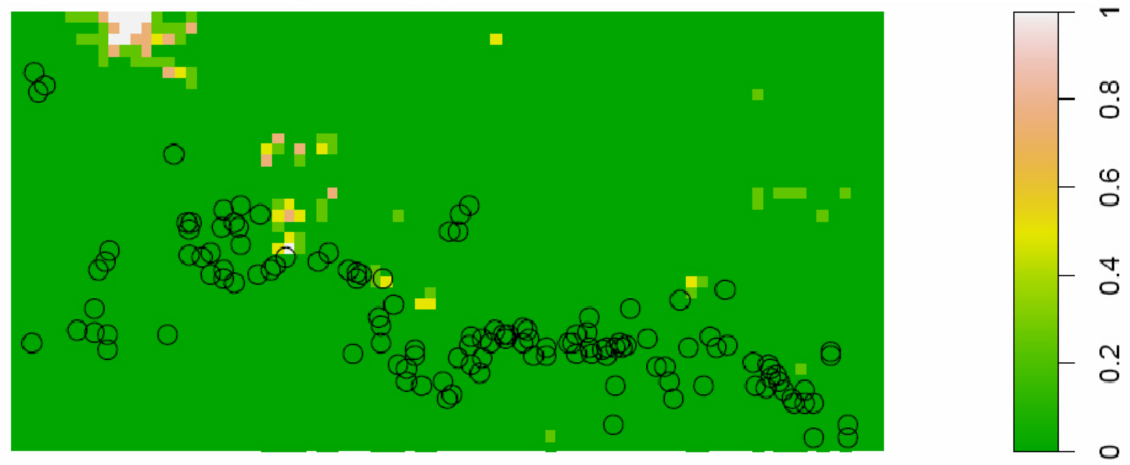

NigUp2

a) *F. nigrescens* (+)

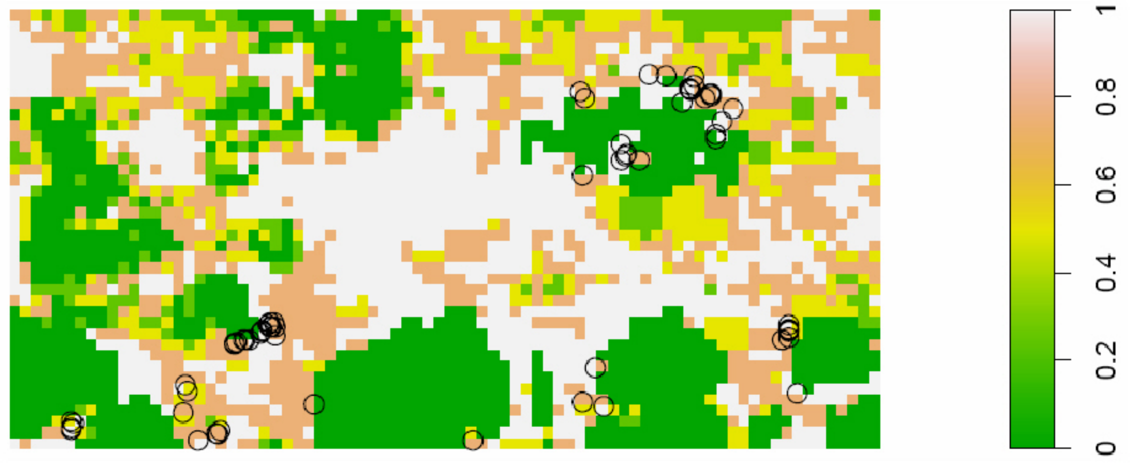

b) *F. eskia* (-)

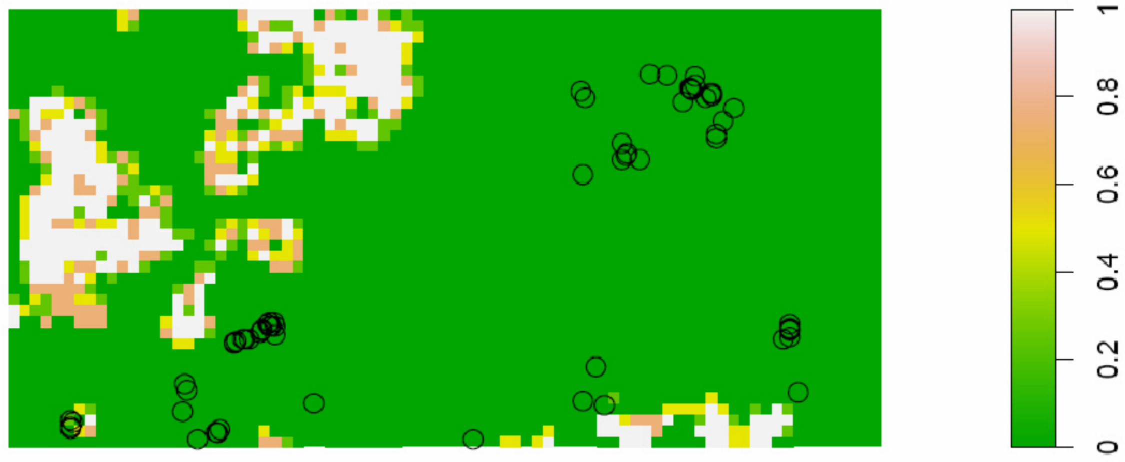

NigDown1

a) *F. nigrescens* (+)

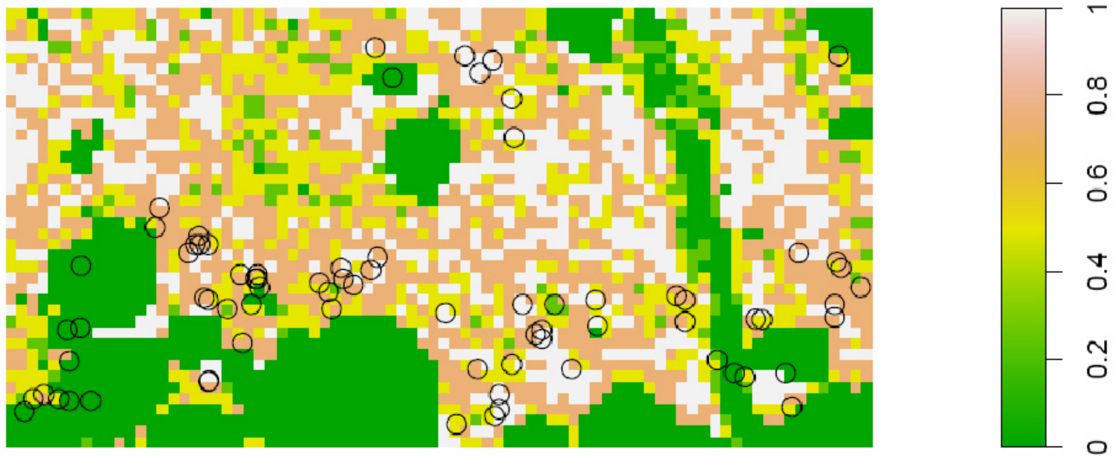

NigDown2

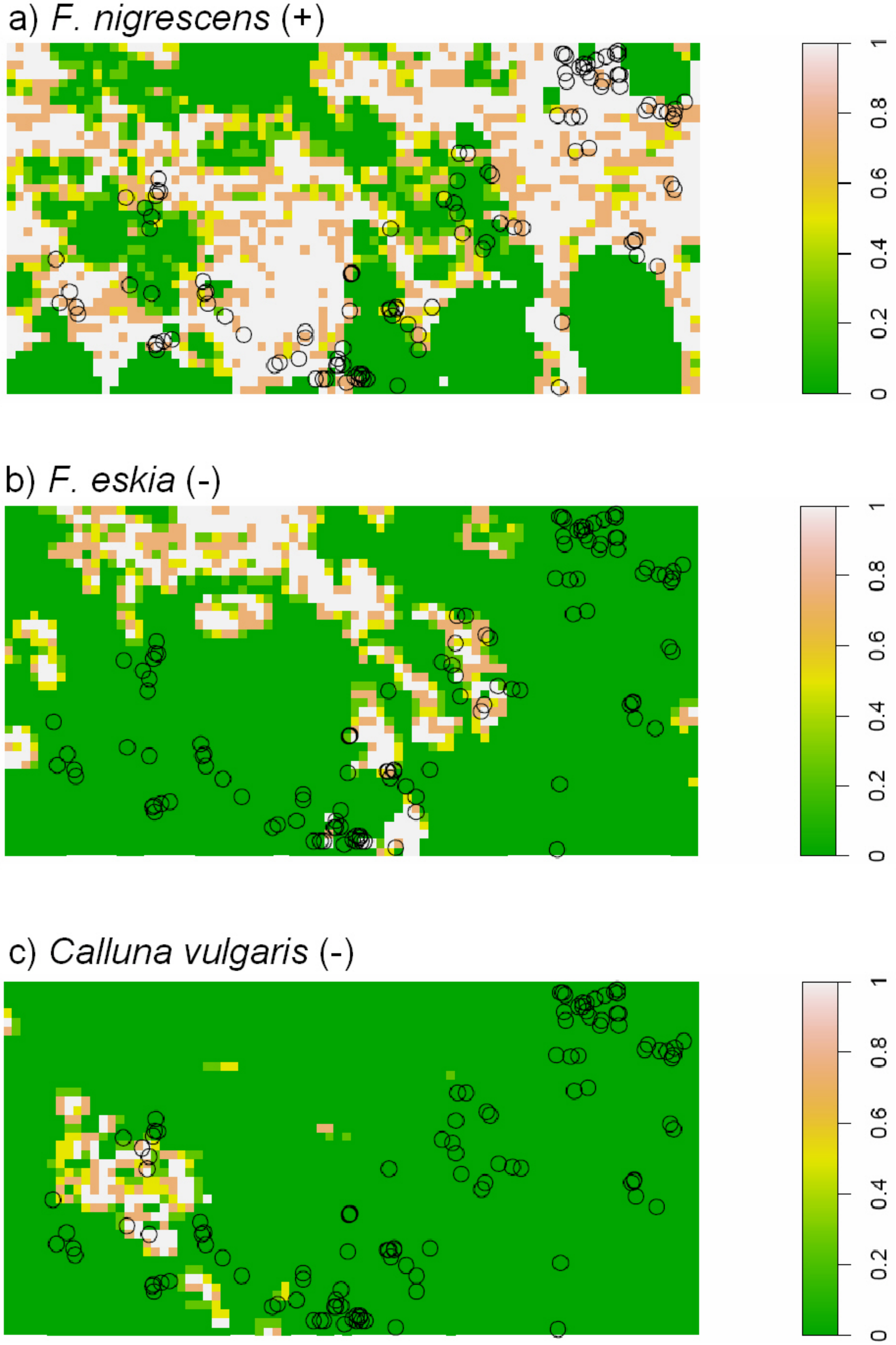

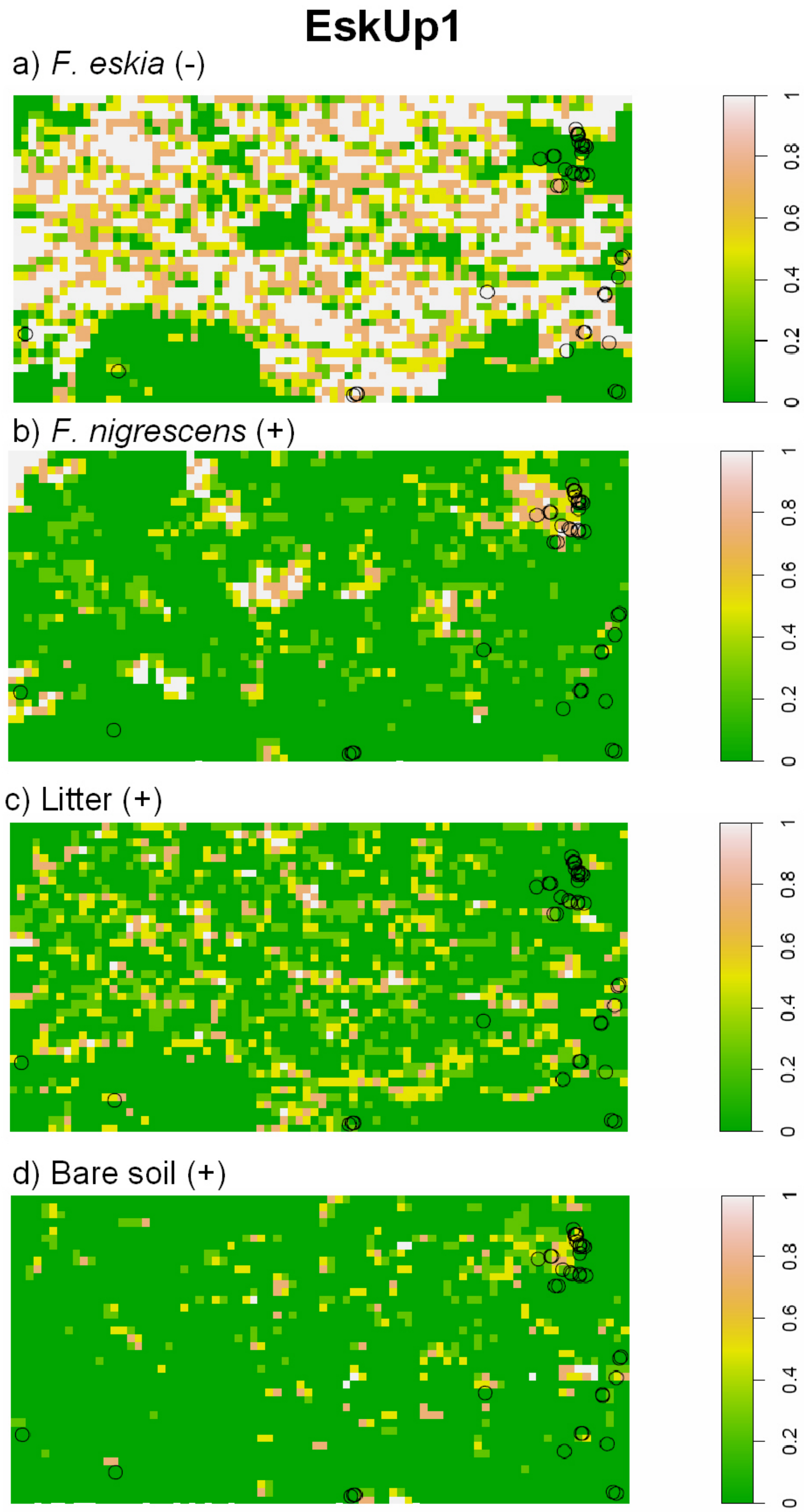
EskUp1
a) F. eskia (-)
b) F. nigrescens (+)
c) Litter (+)
d) Bare soil (+)
0
0.2
0.4
0.6
0.8
1

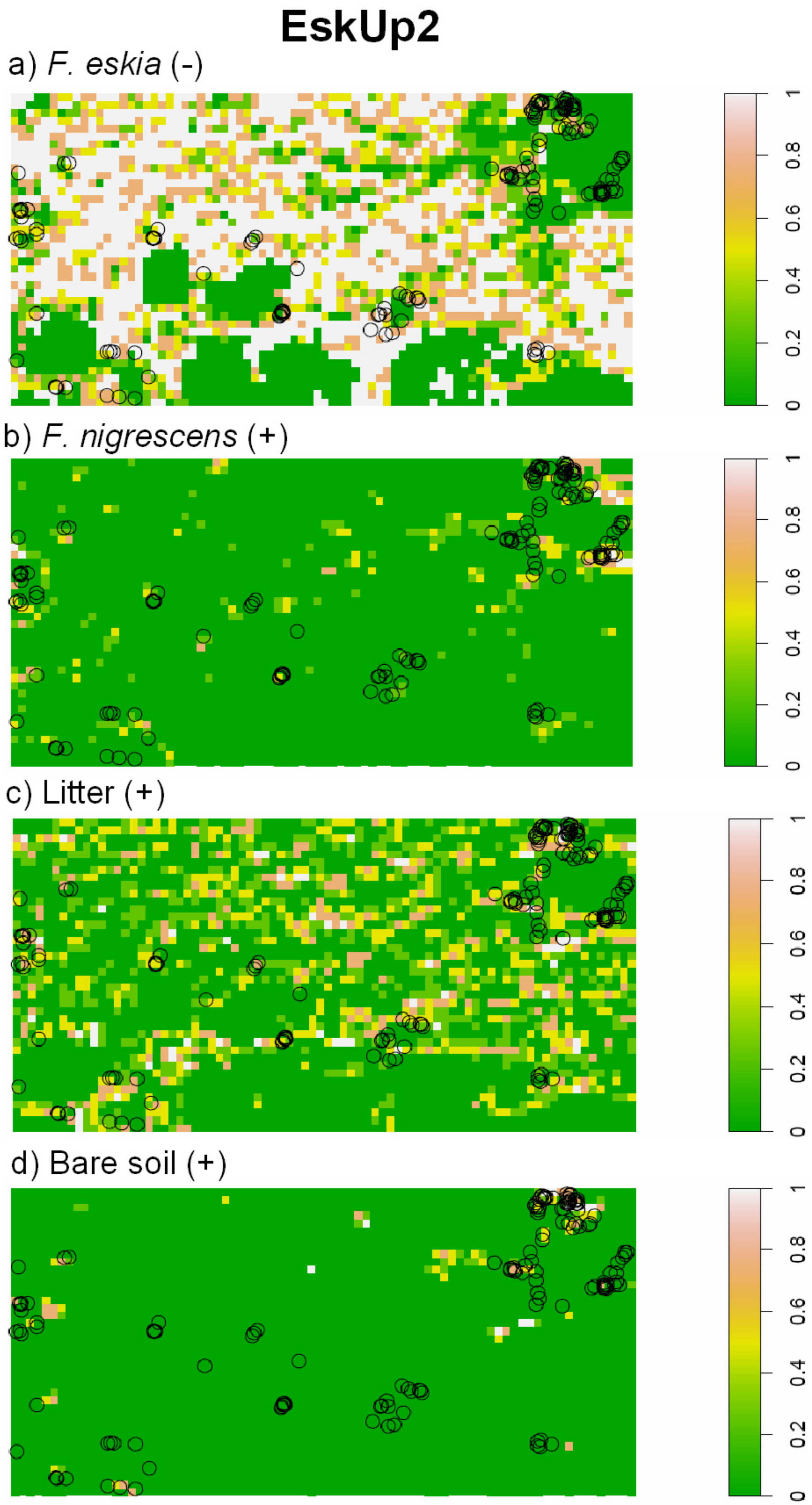
EskUp2
a) *F. eskia* (-)
b) *F. nigrescens* (+)
c) Litter (+)
d) Bare soil (+)

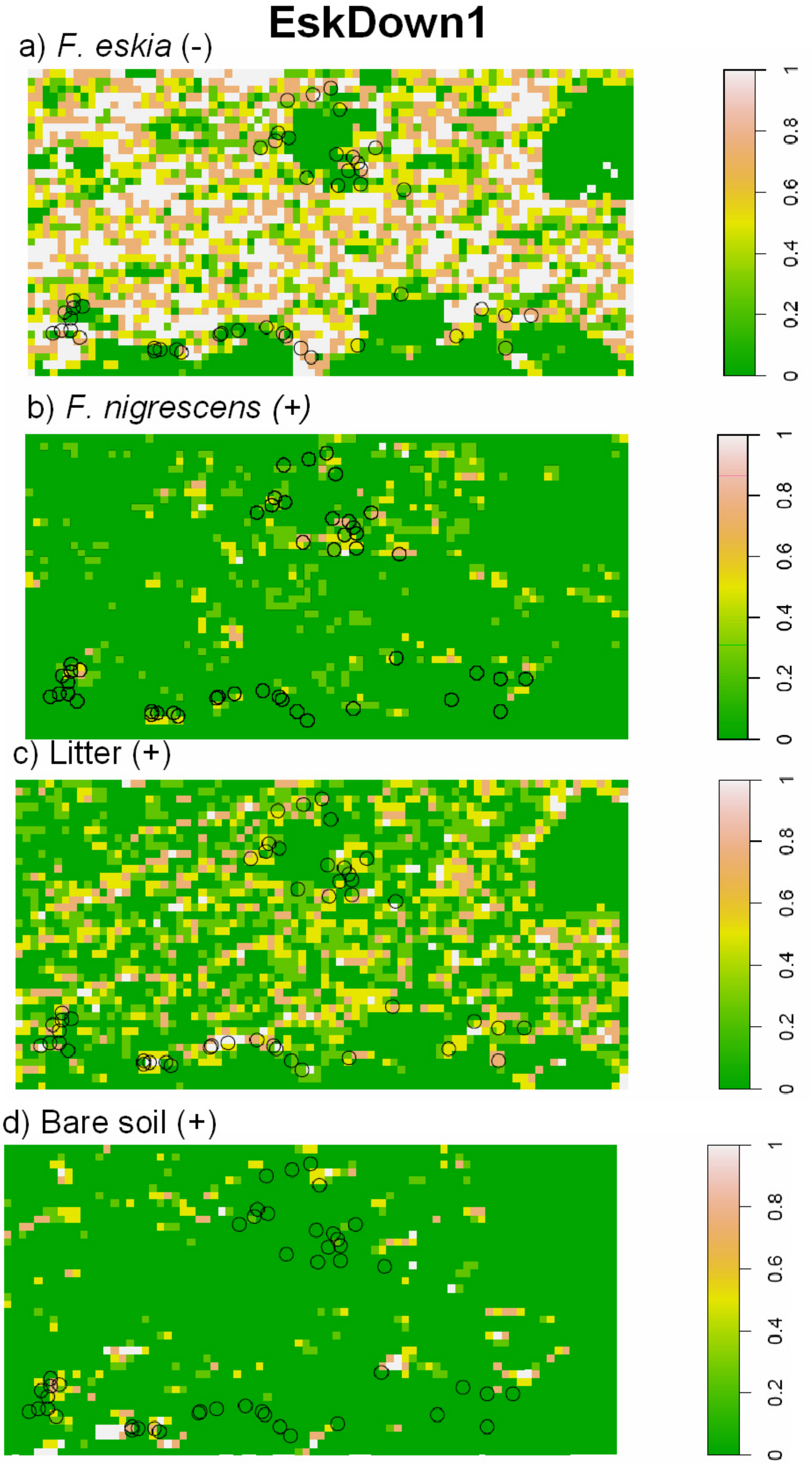
EskDown1
a) F. eskia (-)
b) F. nigrescens (+)
c) Litter (+)
d) Bare soil (+)
0
0.2
0.4
0.6
0.8
1

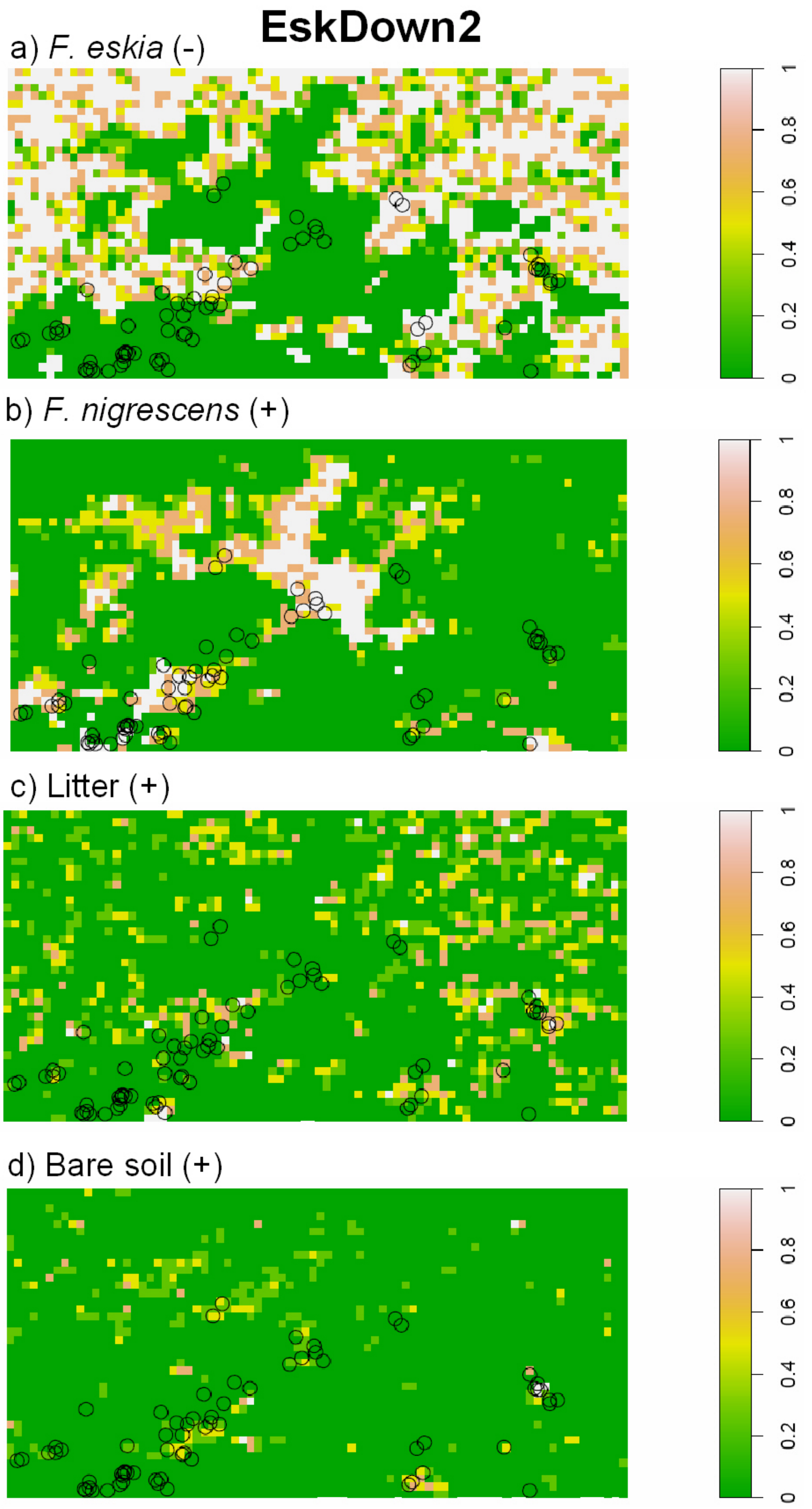
EskDown2
a) F. eskia (-)
b) F. nigrescens (+)
c) Litter (+)
d) Bare soil (+)
0
0.2
0.4
0.6
0.8
1
