# Supplementary figures and images for: How Spatial Heterogeneity of Cover Affects Patterns of Shrub Encroachment into Mesic Grasslands
Source: PLoS One. 2011 Dec 8;6(12):e28652. doi: 10.1371/journal.pone.0028652 (PMC3234287; doi:10.1371/journal.pone.0028652)

## Appendix S2

### a) *Festuca nigrescens* dominated grassland

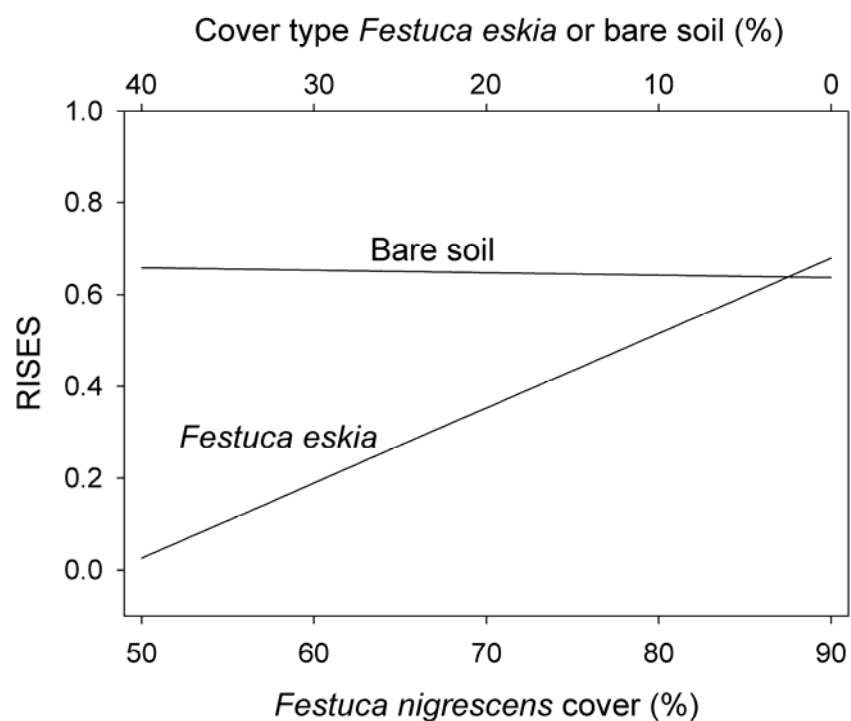

### b) *Festuca eskia* dominated grassland

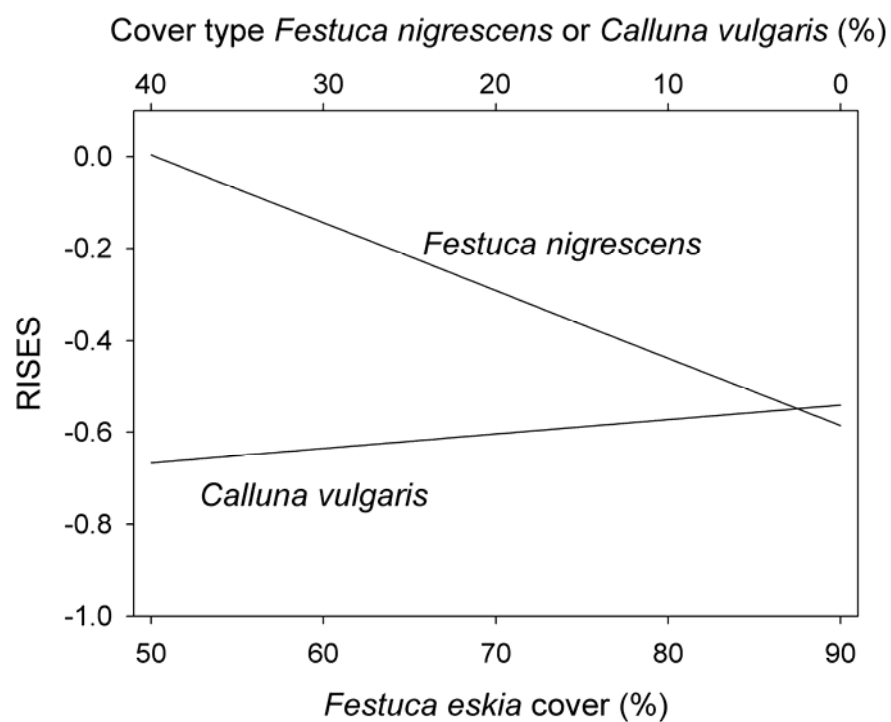

Supplement: Appendix S2 — Changes in RISES values in different grasslands. Changes in RISES values in a) F. nigrescens-dominated grasslands and b) F. eskia-dominated grasslands with dominant species cover ranging from 50 to 90% and an additional represented non-dominant cover type ranging from 0 to 40%. Grassland cover (Cgrass) was assumed to be 100% in all the examples. For each case, an equally-distributed total surface cover of 10% was assumed for the rest of the non-represented cover types (e.g. Carex sp, litter, etc.) in all the situations, and the spatial association values used were derived from our plots. According to the results obtained, changes in surface cover of the dominant grass species are not enough to predict changes in RISES, as RISES values are also dependent on surface cover of non-dominant cover types in both F. nigrescens- and F. eskia-dominated grasslands. (PDF) [file pone.0028652.s002.pdf]
